# Skills to act from a Positive Health approach: in comparison with shared decision-making: a scoping review

**DOI:** 10.3389/fpubh.2025.1530427

**Published:** 2025-05-02

**Authors:** Renske Visser, Martine Veehof, Sandra van Hogen-Koster, on behalf of the RENurse Consortium

**Affiliations:** ^1^Research and Education in Nursing (RENurse), Enschede, Netherlands; ^2^Medical School Twente, Medisch Spectrum Twente, Enschede, Netherlands; ^3^Healthcare Academy, Saxion University of Applied Sciences, Enschede, Netherlands

**Keywords:** Positive Health, shared decision making, healthcare professionals, skills, review

## Abstract

**Introduction:**

Positive Health (PH) is a health approach that expands the definition of health, emphasizing social, psychological, and personal perspectives. PH helps healthcare professionals to provide insight into a patient’s perceived health and gives them insight into their health improvement. PH is acknowledged for improving healthcare and quality of life, but the practical implementation of PH and the necessary skills for healthcare professionals remain unexplored. The overall aim of this review is to explore and map the Positive Health skills needed for healthcare professionals in a variety of healthcare settings.

**Methods:**

A scoping review was conducted. PubMed, Embase, and CINAHL were searched using the key term “Positive Health” AND “healthcare professionals” AND “skills” including synonyms and related keywords. Initial searches yielded fewer relevant studies than expected. Therefore, a revised strategy incorporated “Shared Decision-Making (SDM)” AND “healthcare professionals” AND “skills” to enhance the search. The methodological quality was assessed. A convergent integrated approach synthesized findings and identified overarching skills. An overview was made to visualize the skills.

**Results:**

After screening, 15 studies were included. The five overarching skills are: *“applying a holistic approach”, “communicating and active listening”, “managing time effectively”, “encouraging patient participation”, “reflecting and self-reflection”*.

**Discussion:**

An overview of PH skills was obtained, where the comparison with SDM led to more foundation and more strategies for PH skills. Increasing PH skills in clinical practice may improve implementation. Further research is needed to explore if PH and SDM are mutually reinforcing.

## 1 Introduction

Worldwide, healthcare is under increasing pressure due to an aging population. Longer life expectancy presents valuable opportunities, but its benefits depend on one crucial factor: health (1, 2). Approximately half of the world’s population already lacks access to essential healthcare. Where healthcare is available it is often fragmented and of low quality (1). Moreover, healthcare systems have to manage the increasing diversity of the patient population. This includes both cultural diversity and a broader spectrum of health conditions. In many countries, however, professional education of healthcare professionals does not even meet the demands of this increasingly complex healthcare environment. This lack of education reduces optimal care delivery and limits the capacity to meet diverse healthcare needs (2). An aging population will only exacerbate these challenges, as new diseases may emerge in different patient groups and the number of people with chronic conditions will continue to rise. Existing shortages of healthcare professionals are putting pressure on the healthcare system, making it even more difficult to meet the increasing demands of complex healthcare (1–3).

In response to these challenges, healthcare systems are shifting from systems designed around diseases and health institutions towards systems designed for and with a wide variety of people (1, 2). Moreover, the worldwide perspective on health has evolved from a biomedical model focusing on health and illness, to a concept that emphasizes social and psychological aspects as well as the personal perspective on health.

Person- or Patient-centered care (PCC) is a key component in this new concept of health (1, 4). PCC adopts, respects and responds to individuals’, families’, and communities’ perspectives, needs, values and preferences and promotes them as active participants in their own healthcare (4, 5). Active participation in someone’s own health improves health outcomes, disease prevention and efficient use of healthcare resources and services (4, 6). Various health approaches have adopted the PCC model. Shared decision-making (SDM) is for example a PCC approach, where patients take control of health decisions (7, 8). In a two-way exchange, healthcare professionals guide patients through four phases: presenting treatment options (including no treatment), explaining risks and benefits, exploring patient preferences, and making a joint decision (8, 9). Another example is advanced care planning, where (often palliative) patients express their future goals and wishes early on (7).

Positive health (PH) developed by Huber et al. in 2013 represents a new evolution of PCC and tracks the new concept of health by emphasizing active engagement, resilience and personal empowerment (10, 11). Within the PH approach, Huber defines health as the ability to adapt and take ownership within the physical, emotional and social challenges of life (10). While some studies argue that PH is not radically different from other PCC approaches, Huber’s distinct focus on prevention, lifestyle and overall health—rather than disease—sets it apart, offering broader societal benefits, including cost savings and improved well-being (11). Furthermore, PH is highly multifaceted and applicable across diverse health domains and situations, providing a flexible, shared identity that meets a wide range of needs (10–13).

Positive Health knows six dimensions on which health can be assessed; *bodily functions, mental functions and perception, meaning, quality of life, social and societal participation and daily functioning* (14, 15). These dimensions are seen as the foundation which provides insight into a person’s perceived health and gives them insight into their health improvement. With this approach, healthcare professionals are able to look beyond a person’s illness, focus on strengths rather than weakness, balance the relationship with the patient and refer to the patient’s individual responsibility (10, 14). The dimensions of the PH approach can be used by healthcare professionals to start conversations about an individual’s health and are also visualized in a “spider web diagram”. Therefore, it is also considered as “the alternative dialogue tool” (PH-tool) (12–14). The PH approach can be interwoven throughout any care environment, conversation, or scenario, as long as a person’s resilience, needs, values, preferences, and ownership are accounted for (12, 13).

Although PH is recognized as an effective means of improving health and enhancing quality of life within the new health concept, research remains somewhat limited. While studies highlight the positive impacts of the PH approach, its practical implementation across diverse healthcare settings appears to be insufficiently explored (11–14). Furthermore, little attention is given to the specific requirements, skills or competencies that (future) healthcare professionals need to use the PH approach. Precisely these skills might provide healthcare professionals with guidance on how to use PH more effectively and ensure better implementation, enabling individuals to gain more control over their own health. This emphasizes the importance of reviewing existing literature on PH and obtaining an overview of the skills required in using the PH approach. This review aims to explore and map Positive Health skills needed for healthcare professionals in diverse healthcare settings.

The specific objectives include

1. To explore Positive Health key skills needed for healthcare professionals in diverse healthcare settings.
2. To compare Positive Health skills with Shared decision-making skills to assess the relevance of Shared decision-making skills for Positive Health.
3. To create a foundation and strategies for implementation of Positive Health in diverse healthcare settings.

## 2 Methods

### 2.1 Design

A scoping review was conducted to explore and map a broad overview regarding PH skills for healthcare professionals in diverse healthcare settings. This review is in accordance with the JBI manual for evidence synthesis, and the reporting follows the PRISMA Extension for a scoping review (16, 17).

### 2.2 Information sources

In compliance with three researchers (RV, SK and MV) a search strategy was developed. The key term “Positive Health” AND “healthcare professionals” AND “skills” were combined. Synonyms and keywords were included. Pubmed, Embase and CINAHL were used to search for studies.

In the introduction, we note that there is limited evidence on the practical implications of PH, specifically regarding PH skills. After several search attempts, the researchers concluded that even though there appeared to be enough PH research to write a review, fewer relevant studies specifically addressing PH skills or descriptions referring to PH skills emerged than expected. Therefore, a new specific object (objective 2) and strategy was developed in which Shared Decision-making (SDM) was included. The term “Shared Decision-making” AND “healthcare professionals” AND “skills” were combined with synonyms and keywords in a second search strategy (Appendix A).

SDM was incorporated in the strategy because it has overlapping components with PH. Moreover, prior research suggests that PH and SDM can potentially reinforce each other, making SDM skills relevant to the PH approach (7, 18). Additionally, SDM has been widely researched compared to PH, increasing the likelihood of finding relevant skills and evidence, thereby strengthening the evidence base for PH skills.

In addition to the above search strategy, citation searching was applied in found reviews to identify possible missed studies. Studies were searched, screened and selected in the period from 15 November 2023 to 15 February 2024.

### 2.3 In- and exclusion criteria

#### 2.3.1 Inclusion criteria

Studies were included if they met the following criteria:

- Any qualitative, quantitative, or mixed-method design was used;
- The publication was in English or Dutch;
- The skills studied were intended for healthcare professionals;
- The skills were related to Positive Health or Shared Decision-Making approaches.

#### 2.3.2 Exclusion criteria

Studies were excluded if they:

- Were published more than 10 years ago. Although the original search was broader, this limit was set at 10 years to account for the large body of research available on SDM and the recent increase of studies on Positive Health;
- Lacked a formal study design (e.g., opinion pieces, blogs, conference abstracts, or informal reports).

### 2.4 Selection

After duplicates were removed, studies were selected based on title and abstract by one researcher (RV). For studies that initially met the inclusion criteria full-text studies were gained. Each full-text article was read and checked against the inclusion criteria. The selection procedure and the full-text studies were discussed in research meetings with three researchers (RV, SK, MV), until consensus about the selection of the studies was achieved.

### 2.5 Data extraction

Data was extracted by one researcher (RV) using the JBI mixed methods data extraction form (17). The following data were extracted; author (year), country, focus (PH or SDM) and aim/phenomena of interest, research design, data collection, sample size, setting and analysis. After extracting the key findings of each article, results sections were thoroughly searched and PH and SDM skills were extracted or text fragments and illustrations hinting at PH and SDM skills (Appendix B).

### 2.6 Quality appraisal

A quality appraisal of the selected studies was done in order to assess the relevance, trustworthiness and the results of the included studies. Per study a duo of two researchers (RV or SK or MV) independently assessed the studies using the Joanne Brigss Institute (JBI) appraisal tools (16). Based on the study designs of the included studies a JBI tool was selected. The critical appraisal tool for qualitative studies (19), randomized controlled trials (20), cross-sectional studies (21) and textual narratives (22) were used.

### 2.7 Synthesis

Since qualitative and quantitative studies were included in this study, a convergent integrated approach was used to synthesize the findings (23, 24). When quantitative data was not in textual form, data was sometimes “qualitised,” meaning that quantitative data was extracted and translated into textual descriptions (23). Data was extracted by one researcher (RV). Since it is the main goal of this review to find PH skills, the data of the PH studies were extracted first. The researcher looked for skills, strategies, illustrations and/or text fragments hinting towards skills to perform a PH approach. These textual descriptions were repeatedly examined in which the researcher looked for overarching PH skills on the basis of similarity in meaning. This process was repeated for the SDM studies and hence culminated in overarching SDM skills. These two sets of skills were compared and discussed with three researchers (RV, SK, MV) until consensus was achieved and eventually an overarching set of skills for PH emerged. Moreover, to gain an overview and visualization, the PH and SDM skills were mapped in a model (24).

## 3 Results

### 3.1 Study selection

**Figure 1:**
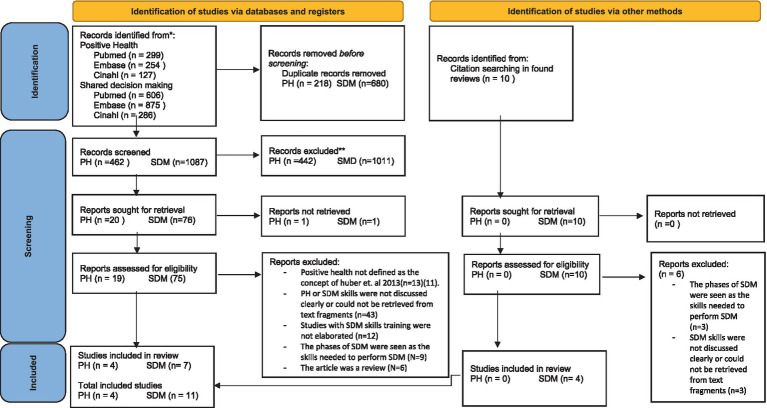
PRISMA flow diagram.

A total (including the PH and SDM search) of 2,447 studies were found in three databases. After removing 898 duplicates, 1,549 studies were screened on title and abstract after which 96 were sought for retrieval. Eventually, 94 full-texts were retrieved, and 10 full-texts were retrieved by citation searching, of which 15 met the inclusion criteria and were included in this review (Figure 1).

### 3.2 Study characteristics

Of the fifteen included studies four studies focused on PH and eleven studies focused on SDM (Appendix B). Studies originated from Netherlands (*n* = 7), Spain (*n* = 1), Germany (*n* = 1), Colombia (*n* = 1), United States (*n* = 3), United Kingdom (*n* = 1) and Taiwan (*n* = 1). Eight studies were based in a hospital setting (25–32), one in a mental health clinic (33), two in a medical center (12, 34), one in an organization for patients with haemophilia (35), one in a general practice (36) and one in a social health organization (37). Most studies had a qualitative design (12, 25, 26, 28–31, 33, 34, 36, 37) (*n* = 11). From these studies PH and/or SDM skills were derived from perspectives, needs or experiences from healthcare professionals (12, 26, 29–31, 33, 37), from healthcare professionals and patients (25, 28) or from patients’ perspectives, needs or experiences with healthcare professionals using PH or SDM (34, 36). Other studies had a quantitative design (32, 35) (*n* = 2). One of these studies examined the effects on attitude and communication of healthcare professionals, specifically when patients had received SDM-training (32). The other study investigated the impact of information provision and attentive listening by healthcare professionals on patients’ perceptions of SDM (35). One study had a mixed-method design (*n* = 1), using surveys and expert interviews with physicians to explore the critical factors of SDM competence (27). The last study differs somewhat from the other designs given that this study was a perspective article and compared health literacy and health portion skills for maintaining and improving PH (38).

### 3.3 Quality assessment

The results of each quality assessment are summarized in two tables (Appendix C). Specifically, two qualitative studies met all quality criteria and are classified as good quality. The remaining studies are categorized as low or moderate quality. However, in consultation with all researchers (RV, SK, MV), all studies remained included, even the poorly scoring studies as the essences of these studies were considered valuable.

### 3.4 Synthesis of results

**Figure 2:**
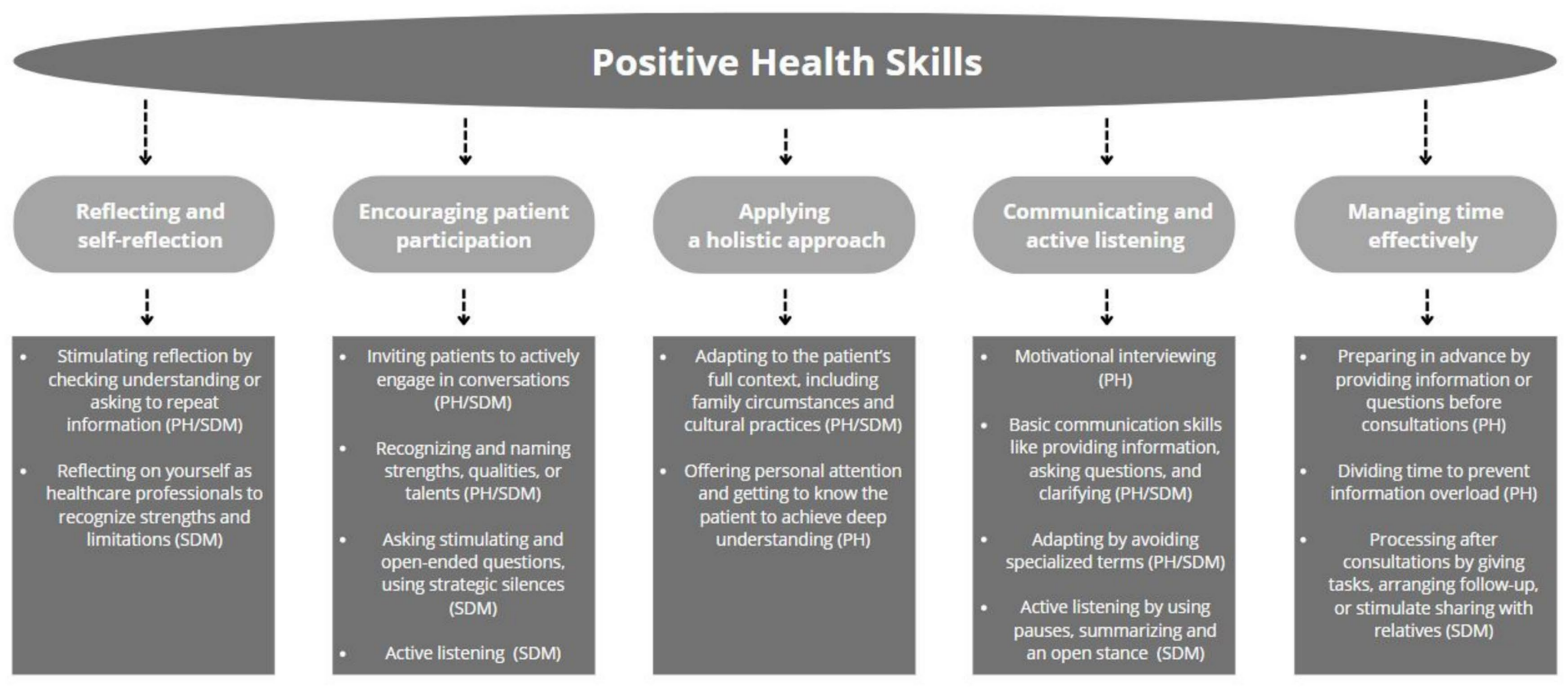
An overview of PH skills and associated strategies.

Several skills, strategies, illustrations and/or text fragments hinting towards skills to perform from a PH or SDM approach have emerged from the studies (Appendix B). After comparison of PH and SDM skills, one set of five overarching skills for PH emerged: *“applying a holistic approach”, “communicating and active listening”, “managing time effectively”, “encouraging patient participation”, “reflecting and self-reflection”*. For each skill, the results reveal what the skill entails and how and to what extent the skill emerged in the PH studies, followed by a comparison with how this skill emerged in the SDM studies. This first of all provides more evidence for the PH skill, as SDM and PH are similar approaches but with the disclosure of differences, it also becomes clear how a PH skill differentiates and needs to be implemented differently. A model was made to gain an overview of the PH skills (Figure 2).

#### 3.4.1 Applying a holistic approach

“Look at patients as a whole” and consequently applying a holistic approach, is viewed as a fundamental skill in establishing trust and a relationship between patients and healthcare professionals and is often seen as a key requirement in conducting PH (12, 36, 38). The PH tool helps provide insight into a patient’s broader context. However, it’s up to the healthcare professional to offer personal attention by getting to know the patient, discovering personal characteristics, and truly understanding the patient (12, 36). This understanding allows for better adaptation of conversations and treatment plans to the patient’s needs, which is important for holistic care (12, 36, 38). PH studies emphasize that effective holistic care requires familiarity with the patient’s full context, including their background, socioeconomic status, health literacy, and family situation for example (12, 36, 38).

Just as PH, SDM requires continuous adaptation to the individual patient. Healthcare professionals adjust the SDM conversation based on the patient’s specific situation, which may influence the decision (25–27, 29, 33, 35). Factors such as family circumstances or cultural practices are considered (25–27, 33). Thus, the context of the patient is taken into account, applying a holistic approach. However, there is a subtle difference. PH studies focus more on personal attention and truly understanding the patient to analyze their overall health context (12, 36). In contrast, SDM emphasizes the healthcare professional’s adaptability to make decisions tailored to specific areas, with less focus on deep patient understanding (25–27, 29, 33, 35).

#### 3.4.2 Communicating and active listening

All PH studies agreed that conducting a PH approach requires a good conversation while conveying genuine attention for each other (12, 36–38). According to most studies, the main emphasis in a PH conversation is on behavior change or goal setting (12, 37, 38). This is because the PH approach gives insight into a person’s health and identifies areas for improvement, utilizing techniques such as motivational interviewing to encourage behavioral changes towards improved health (12, 38). This requires first of all basic communication skills such as providing information, asking questions and clarifying (12, 36, 38). In addition, communication also requires adapting to the patient when speaking to the patient or transferring information, for example, avoiding specialized terms (12, 36–38).

SDM studies, on the other hand, emphasize the importance of being straightforward and precise in a SDM conversation, often referred to as “getting to the point.” This is necessary as SDM conversations often focus on whether and how treatment options are still viable (27, 28, 30, 32, 34). Although one study mentioned the use of motivational interviewing in SDM, behavior change is not seen as a primary goal (32). The communication techniques in SDM are similar to those in PH, but more extensive techniques are mentioned such as using both verbal and non-verbal communication and limiting oneself to only the important information (25, 27–29, 31–35).

However, what emerged in the SDM studies, which was not mentioned in the PH studies, is active or attentive listening. Listening is part of any conversation and is also mentioned in PH studies, but not so much as a separate skill. Active listening is considered crucial in SDM for building trust, helping patients feel heard, and fostering respect (25, 27, 28, 32, 34, 35). This can be accomplished by focusing attention on the patient instead of distractions like a computer, employing nonverbal communication techniques such as an open stance and summarizing the patient’s concerns (27, 28, 34). Additionally, carefully using silences in conversations allows the patient time to reflect and respond, while giving the healthcare provider a chance to listen more deeply and show empathy and understanding (26, 28, 34).

#### 3.4.3 Managing time effectively

Two PH studies stated that the scope of PH or the conversation itself was too time-consuming (12, 36). However, these studies also highlighted that effective time management is necessary. These studies stated that it is the responsibility of healthcare professionals to organize or divide time efficiently so that PH can be followed, and the risk of information overload is reduced. Strategies mentioned included preparation tactics, such as providing information or questions prior to consultations.

The SDM approach was considered as too time-consuming in more than half of the SDM studies (25–27, 29–31, 34). Just as the PH studies, the SDM studies stated that effective time management is essential when conducting an SDM approach. The need for these skills is based on the increasing time pressure and personnel shortages in healthcare, as mentioned by many SDM studies (25–27, 29, 32–34). Whereas the PH studies focus more on time management strategies prior to consultations, SDM studies employed contrasting strategies that focus on processing information after consultations. Some of these strategies include sending patients home with questions, arranging follow-up appointments, and stimulating sharing with relatives or friends (25, 27, 37). Moreover, a few SDM studies mentioned using decision aids to improve patient knowledge and fasten the SDM process (25, 27, 30, 31).

#### 3.4.4 Encouraging patient participation

Prompting, encouraging and empowering are similar commonly used terms in three of the PH studies, and are deployed to keep the patient in control of their health at all times (12, 37, 38). Continuously encouraging patients to participate in the PH process is a very important skill to allow patients to have control over their own health. Encouragement also strengthens and increases patients’ adaptive capacity, confidence, and self-efficacy, contributing to behavioral change (37, 38). Strategies for encouragement include inviting patients to participate in PH conversations, recognizing and verbally naming strengths, qualities or talents and collaborating on identifying relevant activities outside consultations (12, 37, 38). These strategies encourage active participation, better management of health behavior, and following up on health goals outside patient consultation (12, 37, 38).

Many SDM studies echo the PH perspective, emphasizing the importance of keeping patients in control of their decisions (25, 27, 30–32, 35). These studies also see encouragement as an important aspect, contributing to self-efficacy and proactivity (27, 35). In addition, it is mentioned that communication skills such as asking the patient stimulating and open-ended questions, as well as the previously mentioned listening skills, such as dropping a strategic silence to elicit responses or self-reflection, contribute to encouragement (25, 27, 31, 32, 35).

However, a distinction in SDM studies is the challenge healthcare professionals experience in constantly involving the patient in the SDM process. This challenge is due to the experienced difficulties in presenting or discussing all available treatment options to the patient. Healthcare professionals sometimes omit options when patients prefer the healthcare professional to make the decision, when the healthcare professional’s expertise may suggest that certain options are unrealistic, or when patients are easily overwhelmed or confused when presented with too much information or too many choices (25, 29–31, 33, 34).

#### 3.4.5 Reflecting and self-reflection

Being able to self-reflect is a cognitive essential process and seen as a basic skill to improve health (38). The majority of the PH studies named reflection or self-reflection as a valued skill, meaning that healthcare professionals have to stimulate the patient to (self) reflect, to ensure that there is mutual understanding (12, 36, 38). This is illustrated by checking if patients and/or relatives have understood what was explained (36). The PH tool or the PH dimensions can serve as a reflection tool according to one study (12).

Checking if a patient has understood a decision and letting the patient repeat a decision, is also a strategy to reflect in SDM (27, 28). Stimulating the patient to reflect is also widely discussed in the SDM studies (25, 27, 28, 33, 34). However, in addition to getting patients to reflect, SDM studies also emphasized the importance of self-reflection for healthcare professionals. Being aware of one’s own traits, limitations, knowledge, and skills is essential. However, accepting one’s flaws and acting on them is even more crucial as it promotes transparency and trust in healthcare professionals, as well as contributes to the quality SDM (28, 34).

## 4 Discussion and conclusion

### 4.1 Discussion

The general objective of this scoping review was to explore and map PH skills needed for healthcare professionals in diverse healthcare settings. As part of this process, PH key skills were identified, compared with SDM skills, and strategies examined for implementation. This structured approach allowed us to gain deeper insights into the practical application of PH in different healthcare settings. To our knowledge this is the first review to obtain an overview of PH skills needed for healthcare professionals.

Five overarching PH key skills were identified from the studies; *“applying a holistic approach”, “communicating and active listening”, “managing time effectively”, “encouraging patient participation”, “reflecting and self-reflection”*. By comparing PH skills with SDM a stronger foundation was given to PH skills and additional strategies provided. While the basics of PH and SDM skills were similar, differences arose in the purpose or application of specific skills. First *“applying a holistic approach”*, was necessary in both approaches, emphasizing the need for a trusting relationship as the basis for PH and SDM (12, 25, 28, 29, 33, 35–38). PH studies placed more emphasis on really understanding a patient to start a meaningful PH-dialogue (12, 36, 38). *“Communicating and active listening”* were central to both approaches and conversations, though SDM studies placed more emphasis on listening skills (12, 25–29, 31–38). *“Encouraging patient participation”* and *“reflecting and self-reflection”* were also key in conversing, empowering patients to take control of their health while achieving mutual understanding (12, 25, 27, 28, 30–38). Self-reflection as mentioned in SDM studies further enhances the quality of these conversations by highlighting the healthcare professional’s role (28, 34). Lastly, *“managing time effectively”* is seen as essential in both approaches for reducing time pressure, preventing information overload, and aligning with patients’ needs (12, 25–27, 32, 34, 36–38).

The differences between PH and SDM skills, as briefly mentioned, can be largely explained by the limited PH research. Since more SDM studies were included in our review, they naturally offer more detailed skills, explanations and strategies. For example, in the skill *“communicating and active listening”* active listening is highlighted in SDM studies, but not in PH studies (25, 27–29, 31–35). While listening is part of any conversation, the PH studies did not elaborate on proper listening techniques. Similarly, in *“reflecting and self-reflection”* PH studies acknowledge the need to reflect on the PH approach, but are less specific on self-reflecting, which is discussed in the SDM studies (12, 25, 27, 28, 33, 34, 36, 38). These small differences in techniques or strategies appear across most skills, though the main goal—whether it’s listening, reflecting, or encouraging patients—remains mostly the same. Since both approaches pursue similar goals, it is likely that techniques or strategies are applicable to PH, and vice versa.

However, there are three notable differences of which two reside in the focus of conversation. First, in the skill *“communicating and active listening”* came forward that the focus of a PH conversation is to achieve behavior change which is probably less fitting for SDM (27, 28, 32, 34, 36–38). Despite having the same communication and listening techniques, the ultimate purpose of SDM is to reach a decision, which is accomplished by following specified phases (39, 40). It is thus critical to be straightforward and explain exactly what options exist in order to reach a decision. Furthermore, SDM is often applied in a decision in which the extent of evidence and quality varies, often making it a complex decision (9). It is therefore vital to communicate clearly and get to the point of the conversation quickly in order for patients to understand their position. When looking at the PH approach, the ultimate purpose is to obtain understanding into a person’s health by broadening and deepening six health dimensions, with the individual or patient at the center (14, 15). In partnership with the healthcare professional, the patient considers what is essential for their own health, consequently the patients’ characteristics, values, and preferences regarding their own health will be uncovered (12, 13). The alternative dialogue will also highlight areas for development, and the patient, in collaboration with the healthcare provider, will eventually seek intrinsic motivation in order to enhance their health (13, 14). Consequently, strategies such as motivational interviewing and goal setting are appropriate. Moreover, this also explains the second difference between PH and SDM, as PH studies placed more extensive emphasis on *“applying a holistic approach”* compared to the SDM studies, since a deeper understanding is essential for achieving behavior change (12, 13, 36). While the SDM approach is adapted to the individual and does invest in understanding the person, it does so to a lesser extent (25–27, 29, 33, 35).

The last difference seems to be more of a barrier in the SDM approach mentioned in *“encouraging patient participation”.* While most SDM studies emphasize patient involvement in decision-making, they highlight challenges in reviewing all treatment options. Patients often believe the healthcare professional knows best, and professionals may limit information to avoid overwhelming them (25, 29–31, 33, 34). This barrier, confirmed by other studies, contributes to poor SDM implementation (8, 41). In general, both PH and SDM face implementation challenges. PH struggles likely due to limited research, while SDM faces barriers such as time constraints, lack of decision-making tools, negative beliefs about SDM or overly medical specialized roles (8, 25, 34, 41, 42). Though not directly mentioned in PH studies, similar barriers are likely, as confirmed by one study included in our review that stated that the scope of PH is quite broad and too time-consuming (12). Addressing these barriers is critical to the successful implementation of PH or SDM in diverse healthcare settings, as they can hinder patient participation and erode trust in the patient-professional relationship. By identifying PH key skills and comparing them to SDM, our research not only strengthens the foundation for PH, but also provides concrete strategies for these barriers and to facilitate its implementation.

Another perspective that could enhance the implementation of both approaches and help overcome barriers is the potential for PH and SDM to reinforce each other, given their many similarities identified in our review. Pel et al. (7) and Hofman et al. (18) attempted to provide insight into how PH and SDM or other methods based on PCC enrich each other and confirm the similarities found between PH and SDM in our review. By including the open approach of PH in SDM, all aspects of health by which the decision is affected, are considered. Patients and healthcare professionals are encouraged to weigh the options of the decision against the different areas of health and thus also make more thorough considerations. This can result in a decision more tailored to the individual which goes beyond medical treatment and perhaps lead to better relationship building (7, 18). When looking at how SDM can strengthen PH, it is especially apparent that clinical expertise is still sometimes missed in PH conversations (12). This highlights precisely the aspect that emerges positively from SDM and can be effectively applied in PH practices, further supporting its implementation in various healthcare settings.

#### 4.1.1 Strengths and limitations of the study

This review presents both strengths and limitations. The majority of our included studies had a qualitative design, this is not only appropriate but also logical and valuable in relation to the topic and objective of the study. Since PH and SDM are approaches that focus on the meaning individuals assign to their health or the significance they attribute to decision-making, it is entirely natural that the included studies focus on qualitative aspects. This emphasis on qualitative research is considered a strength, as it offers in-depth insights into experiences and perspectives and enabled a rich and nuanced exploration of healthcare professionals’ PH and SDM skills. Although the primary purpose of our review was qualitative in nature, we included quantitative studies because of the limited literature available. These quantitative findings were translated into textual descriptions to better capture the underlying meanings and ensure consistency with the qualitative data.

Additionally, there are limitations related to the JBI appraisal tools used for assessing study quality. First, a qualitative appraisal tool was used for a mixed-methods study due to the absence of a mixed-methods JBI tool. Although the study was mostly quantitative, our review focused on its qualitative data, making the tool appropriate for a rigorous evaluation. Furthermore, only two studies achieved a maximal JBI score, categorizing them as high quality, while the remaining studies were rated as having low to moderate evidence. Ambiguity often arose in the qualitative studies due to the researcher’s philosophical perspective not aligning clearly with the chosen research methodology. Including these studies may be viewed as a limitation; however, their fundamental findings were universally deemed valuable by all researchers. Moreover, PH research is limited, and without these studies, a review would not have been possible.

Given the limited scope of PH research, the initial methodology of the review—to only use PH studies—was quickly adjusted. We decided to include SDM studies, which is a strength because it provided a better comparison, more substantial evidence and more applicable PH skills. Ultimately, however, only four PH studies were included, providing an uneven and thus potentially inferior comparison, resulting in a possible limitation. Therefore, the comparison between SDM and PH skills should be approached with caution. Moreover, while our study found many similarities between the two, there are also some differences. PH and SDM remain different approaches, each with a different purpose. Thus, using the skills interchangeably does not seem entirely feasible. Moreover, more evidence is needed to determine whether PH skills can be effectively integrated into SDM and vice versa.

Another potential limitation is that our review did not encompass other PCC methods, such as advanced care planning or PROMs, which might have identified additional implementable skills. At the same time, adding SDM to the review has not led to new additional skills, only refinements of the PH skills.

The inclusion of SDM studies significantly expanded the available research. As a result, studies older than 10 years were excluded, which may have led to the omission of relevant SDM studies. Including only eleven SDM studies might also seem limited given the amount of available research. However, similar to the selection of PH studies, a thorough and precise selection process was applied. Only studies providing a comprehensive description of skills or offered a large amount of text fragments hinting towards skills were included. Studies were excluded, for example, if they merely mentioned “communication” as a skill without explaining how to communicate or if they treated the phases of SDM as separate skills.

### 4.2 Conclusion and practice implications

In conclusion, this scoping review mapped and provided an overview of the PH skills needed for healthcare professionals in diverse healthcare settings. In achieving this, the following conclusions can be drawn:

1. Five overarching PH key skills essential for healthcare professionals across diverse healthcare settings were identified, offering a comprehensive understanding of the skills needed in practice.
2. By comparing PH skills with SDM skills, more foundation was provided for the PH approach. This comparison not only highlighted similarities but also identified relevant strategies that can be used to further develop and apply PH skills in diverse healthcare settings.
3. Based on the identified PH skills and the comparison with SDM, more foundation and strategies for the implementation of PH in healthcare settings were established. This can serve as a guideline for healthcare professionals aiming to integrate PH into their practice.

These findings highlight the need for further research on the practical application and implementation of PH in healthcare. Quantitative research on the effectiveness of these skills at scale, would be valuable in assessing their broader applicability and impact in different healthcare settings. Practical training to improve the PH skills of (future) healthcare professionals could improve implementation, resulting in improved health, well-being and active participation of patients. In addition, combining PH with SDM could improve the effectiveness of both approaches. Although some studies examine this combination, most are based on assumptions and lack practical evidence. More research on implementing PH and SDM together would strengthen the evidence and improve the quality of care.

## Group member of RENurse consortium

Elze Bent, Annemarie de Vos, Jeroen Dikken, Monique Dings, Catharina van Oostveen, Hester Vermeulen, and Nadia Segers.
