# Supplementary material for: Skills to act from a Positive Health approach: in comparison with shared decision-making: a scoping review
Source: Front Public Health. 2025 May 2;13:1530427. doi: 10.3389/fpubh.2025.1530427 (PMC12081412; doi:10.3389/fpubh.2025.1530427)
Supplement: Supplementary file 1 [file Table_1.docx]

**Pubmed**

| **Search terms** | **Search string** |
| --- | --- |
| Health care professionals | (((Health [tiab] OR healthcare [tiab]) AND (professional* [tiab] OR personnel [tiab] OR provider* [tiab] OR worker* [tiab])) OR Nurs* [tiab] OR Physician* [tiab] OR Paramedic* [tiab] OR Therapist* [tiab] OR Health Personnel [mesh]) |
| Positive Health  Shared decision making | AND (Positive health [tiab] OR PH-tool [tiab] OR Positive Health Tool [tiab] OR Health dimension* [tiab] OR My positive health tool [tiab] OR MPH tool [tiab] OR Pillar* Positive Health [tiab])  AND (Shared decision making [tiab] OR shared decision-making [tiab] OR decision making, shared [mesh]) |
| Skills | AND (Competency [tiab] OR Competencies [tiab] OR skill* [tiab] OR communication [tiab] OR attitude [tiab]) |

**Embase**

| **Search terms** | **Search string** |
| --- | --- |
| Health care professionals | ('Health care professional':ti,ab,kw OR 'Health personnel':ti,ab,kw OR 'Health care provider*':ti,ab,kw OR 'Health care worker*':ti,ab,kw OR 'Health professional*':ti,ab,kw OR 'Nurs*':ti,ab,kw OR 'Paramedic*':ti,ab,kw OR 'Healthcare professional*':ti,ab,kw OR 'Healthcare provider*':ti,ab,kw OR 'Healthcare worker*':ti,ab,kw OR 'Health professionall':ti,ab,kw OR 'Health provider*':ti,ab,kw OR 'Health worker*':ti,ab,kw OR 'Therapist':ti,ab,kw OR 'health care personnel'/exp OR 'paramedical personnel'/exp OR 'nurse'/exp) |
| Positive Health  Shared decision making | ('Positive health':ti,ab,kw OR 'PH-tool':ti,ab,kw OR 'Positive Health Tool':ti,ab,kw OR 'Health dimension*':ti,ab,kw OR 'My positive health tool':ti,ab,kw OR 'MPH tool':ti,ab,kw OR 'PH dialogue tool':ti,ab,kw OR 'Pillar* Positive health':ti,ab,kw)  ('Shared decision making':ti,ab,kw OR 'shared decision-making':ti,ab,kw OR 'shared decision making'/exp) |
| Skills | ('Competency':ti,ab,kw OR 'Competencies':ti,ab,kw OR 'Skill*':ti,ab,kw OR 'Communication':ti,ab,kw OR 'Attitude':ti,ab,kw 'competencies'/exp) |

**Cinahl**

| **Search terms** | **Search string** |
| --- | --- |
| Health care professionals | (MH "Health Personnel+") OR TI ("Health care professional*" OR "Health personnel" OR "Health care provider" OR "Health care worker" OR "Health professional*" OR "Nurs*" OR "Paramedic*" OR "Healthcare professional*" OR "Healthcare provider*" OR "Healthcare worker*" OR "Health professional*" OR "Health provider*" OR "Health worker*" OR "Therapist*")  OR AB ("Health care professional*" OR "Health personnel" OR "Health care provider*" OR "Health care worker*" OR "Health professional*" OR "Nurs*" OR "Paramedic*" OR "Healthcare professional*" OR "Healthcare provider*" OR "Healthcare worker*" OR "Health professional*" OR "Health provider*" OR "Health worker*" OR "Therapist*") |
| Positive Health  Shared decision making | TI ("Positive health" OR "PH-tool" OR "Positive Health Tool" OR "Health dimension" OR "My positive health tool" OR "MPH tool" OR "PH dialogue tool" OR "Pillars Positive Health") OR AB ("Positive health" OR "PH-tool" OR "Positive Health Tool" OR "Health dimension" OR "My positive health tool" OR "MPH tool " OR "Pillars Positive Health")  (MH "Decision Making, Shared+") TI ("shared decision making") OR ("shared decision-making") OR AB ("shared decision making") OR ("shared decision-making") |
| Skills | TI ("Competency" OR "Competencies" OR "Skill*" OR "Attitude" OR "Communication") OR AB ("Competency" OR "Competencies" OR "Skill*" OR "Attitude" OR "Communication") |
