# Supplementary material for: Skills to act from a Positive Health approach: in comparison with shared decision-making: a scoping review
Source: Front Public Health. 2025 May 2;13:1530427. doi: 10.3389/fpubh.2025.1530427 (PMC12081412; doi:10.3389/fpubh.2025.1530427)
Supplement: Supplementary file 2 [file Table_2.docx]

| Author, title and country | Focus (PH or SDM) and aim | Design | Data collection/ sample size/ setting | Analysis | Key findings | Found skills |
| --- | --- | --- | --- | --- | --- | --- |
| Droes et al. (2017)  Social health and dementia: a European consensus on the operationalization of the concept and directions for research and practice  Netherlands | Positive health  To operationalize the new health concept for people with dementia, specifically the social domain, and to formulate directions for research and practice to promote social health in dementia | Qualitative | Operationalizing three social health dimensions with three groups existing of social health members (n=54) | Surveys and consensus meetings. Reviewed by the European Working Group of People with Dementia | Personal, disease-related and environmental factors that can influence the person with dementia’s social health and many interventions promoting social health were identified | **Communication and active listening**   - Support adapting and cope with changing abilities and limitations - Promote capacity: use talents, use technologies to social inclusion,   **Encouraging patient participation**   - Building capacities, use of talents, use of assistive technologies related to social inclusion and advanced care planning - Promote capacity: use talents, use of strengths, use technologies to social inclusion, - Enhance resilience: promote adaption, utilizing personal and social assets, overcoming consequences of the disease - Enhance a person’s strengths and capabilities - Promoting participation in social activities/ stimulate sharing with others - Use different interventions such as goal-oriented change - Support adapting and cope with changing abilities and limitations |
| Rimmelzwaan et al. (2020)  Multimorbidity in General Practice: Unmet Care Needs From a Patient Perspective.  Netherlands | Positive Health  To explore the general practice care needs of patients with multimorbidity, their care experiences and the impact of chronic conditions on a patients daily functioning | Qualitative | Semi-structured interviews with patients from general practices having three or more chronic conditions (n=12) | Thematic analysis with constant comparison | Participants were positive about the relationship with the general practitioner and practice nurse as well as the care they received. Unmet care needs were: lack of a holistic approach, lack of personal continuity of care, lack of patient-tailored explanations about diseases and treatments | **Applying a holistic approach**   - “look at patients as a whole” - Giving personal attention - “Get to know them” - Treat patients as equals - Need for a holistic approach - Give more attention to multiple chronic patients - Personal continuity of care needed, care needs based on their functioning - Build an open relationship: reliable, approachable and concerned with the patient   **Communication and active listening**   - Adapt explanations to the level of understanding - “my general practitioner tries to explain everything clearly to me, but he does not write it down” - Sometimes only medical jargon - Make explanations more tailored to their level of comprehension - Personal attention in conversation   **Managing time effectively**   - Increased time pressure and personnel shortage leads to having time for one item and less time for patients - “my general practitioner tries to explain everything clearly to me, but he does not write it down”   **Reflecting and self-reflection**   - Check sufficiently with the patients if everything is understood |
| Bock et al. (2021)  Positive Health dialogue tool and value-based healthcare: a qualitative exploratory study during residents' outpatient consultations  Netherlands | Positive health  To explore how residents experienced the application of the Positive Health tool (PH-tool) during outpatient consultations and its influence on the delivery of value-based healthcare (VBHC) | Qualitative | Observations of residents (n=8) having consultations with patients (n=79) using the PH-dialogue tool in a university medical centre. | Iterative-inductive thematic approach | The main benefits according to residents using the PH-tool: insight in the patients’ individual context, changed dynamics in communications, increased awareness regarding value in terms of patient-related outcomes and healthcare costs. Three barriers: doubt regarding the PH-tool’s relevance and scope, boundaries of super specialised medical professionals, and a lack of demarcation in clinical practice | **Applying a holistic approach**   - Customize treatment plan to individual patient - Use PH tool for health changes and leads to more talkative attitude and conveyed more personal information   **Communication and active listening**   - Prompting patients to engage in the conversation - Not always enough clinical expertise during PH conversation   **Managing time effectively**   - Giving the tool/information prior to the consultation - Making more time - PH tool contained too many statements, sometimes overlapping, hereby too time consuming to discuss everything during encounter   **Encouraging patient participation**   - Prompting patients to engage in the conversation - Probing patients preferences into rationales, PH tool helps - Use PH tool for health changes and leads to more talkative attitude and conveyed more personal information   **Reflecting and self-reflection**   - Encourage patients self-reflection - Use PH tool as reflection tool |
| Sponselee et al. (2023)  Letting people flourish: defining and suggesting skills for maintaining and improving positive health  Netherlands | Positive health  Defining and suggesting skills for maintain and improving positive health. | Perspective article | Using a functional, interactive and critical health literacy framework. Focusing on skills for patients | Comparison with health literacy and health portion interventions. | A description of specific skills by describing health literacy, executive functions and life skills | **Applying a holistic approach**   - “Diversity of patients requires a broader view and more time because a tailored approach is needed” - Low social economic position, more vulnerable, more adaption needed   **Communication and active listening**   - Health literacy professionals skills such as conversing and consulting - Use behaviour change techniques - Motivational interviewing: formulating implementation intentions, goal setting, setting graded tasks, planning coping responses   **Managing time effectively**   - “Diversity of patients requires a broader view and more time because a tailored approach is needed”   **Encouraging patient participation**   - “identify all relevant skills they (patients) want to work on” - “discuss with clients which skills they want to work on” - Participation in health is starting point for behaviour change - “determine on which skills it is meaningful and feasible to intervene” - Help to understand one’s own health and encourage participating contributes to self-efficacy - Stimulate patient to ask questions, contributes to confidence   **Reflecting and self-reflection**   - The cognitive process of self-reflection is also essential as a basic skill to improve health - Stimulate self-reflection |
| Del Río-Lanza et al. (2016)  Information provision and attentive listening as determinants of patient perceptions of shared decision-making around chronic illnesses.  Spain | Shared decision making  To model the impact of provision of information and attentive listening on perceptions of SDM | Quantitative  Survey | Patients with haemophilia (N=181) From Spanish Federation of Haemophilia and regional haemophilia  Organisations. | Correlations analysis and confirmatory factor analysis | A model was designed. Results show that provision of information and attentive listening determine PPSDM through patients’ self-  efficacy and proactivity in requesting information. | **Applying a holistic approach**   - Take personal characteristics into account (gender, health literacy, severity of the illness, self-advocacy)   **Communication and active listening**   - Establish a collaborative dialogue - Provision of information - Attentive listening: listen actively, for example summarizing the patient’s beliefs and concerns. à creates respect, feeling important, trust - Attentive listening environment is a free and open exchange à improves relationship, two way communication   **Encouraging patient participation**   - Encourage patients self-efficacy and proactivity |
| Hamann et al. (2017)  Training patients with schizophrenia to share decisions  with their psychiatrists: a randomized-controlled trial  Germany | Shared decision making  To evaluate the effects of a patient directed SDM-  training on patients’ communicative behaviour in the consultation, their attitudes towards decision-making and their long-term adherence. | Quantitative  Randomised control trial | N = 264 patients were recruited in four psychiatric hospitals in Germany, divided into two groups, undergoing a SDM training. | Group differences regarding the main outcome parameter (adherence) were analyzed using a Chi2-test | While there was no effect regarding treatment  adherence, the shared decision-making training for inpatients with schizophrenia has been shown to increase  patients’ active behaviour in psychiatric consultations during their inpatient treatment. | **Applying a holistic approach**   - Adapt to the individual situation   **Communication and active listening**   - Communication skills (asking questions, information provision, being assertive) - Motivational interviewing   **Managing time effectively**   - Patients preparing for consultations or asking for treatment alternatives - Implementing decision support tools   **Encouraging patient participation**   - Empower patients to strengthening patients’ abilities to demand participation in treatment decisions |
| Moleman et al. (2020)  Shared decision‐making and the nuances of clinical work: Concepts, barriers and opportunities for a dynamic model  Netherlands | Shared decision making  To describe how healthcare professionals manoeuvre the nuances of decision-making that shape SDM, barriers were identified, and strategies were collected to help healthcare professionals think beyond existing solution pathways and overcome barriers of SDM | Qualitative | Semi-structured interviews with healthcare professionals (n=68) and patients (n=15) were performed from different departments of a hospital | Thematic analysis | Healthcare professionals conceptualize SDM in different ways, which indicates a lack of consensus about its meaning. We identified five barriers that limit manoeuvring space for SDM and contest the feasibility of a uniform, normative SDM model | **Applying a holistic approach**   - “your approach of SDM will depend on a variety of things, patient’s age, the expectations of the physician, the severity of the disease, the diagnosis” - “Asking patients how they feel about the advised treatment and whether it fits into their life”   **Communication and active listening**   - Building a trusting relationship à active listening and adapting the communication style - Procedural justice strategies: allow room for patients to object or respond à fostering a bilateral partnership   **Managing time effectively**   - Mentally prepare patients for SDM for example by summary of the patients status - Follow up: send patient home with specific questions - Use online tools such as decision aids - Involve an independent party to have a time out conversation   **Encouraging patient participation**   - Encourage/invite patients to participate à “what are your expectations”, “what do you want - Assure that patients make the final call - Instruct the patients about their rights - Give a sense of control, even in the little choices   **Reflecting and self-reflection**   - Checking understanding - Strengthening the reflective ability à for example use a tool - “Asking patients how they feel about the advised treatment and whether it fits into their life” - Not all patients want new roll in SDM |
| Whitney et al. (2021)  Trust and shared decision-making among individuals with multiple myeloma: A qualitative study  America | Shared decision making  To shed light on the development of trust between multiple myeloma patients and clinicians, as trusting relationships are essential to promote effective SDM, that aligns with clinical practice with patients values and preferences | Qualitative explorative | Semi-structured interviews with MM patients (N=19) in academic or community based medical centers | Inductive thematic | Many factors contribute to the development of trust, some are outside of clinicians’ control, others drive from clinician behaviours and interpersonal communication skills | **Applying a holistic approach**   - Be reliable to promote trust for example by: following up with abnormal results, returning patients phone calls - Involve empathy and warmth in communication   **Communication and active listening**   - Be willing to spend time listening/actively listening - Be honest and transparent in communication, by for example given a direct explanation of the disease, be detailed - Involve empathy and warmth in communication - Be informed and knowledgeable   **Managing time effectively**   - Be willing to spend time listening/actively listening - Focus on the patient, not the computer   **Encouraging patient participation**   - Give reassurance when a patient seeks out knowledge - “I want to hear their explanations about each option”, physicians are not always aware of each option |
| Luna-Meza et al. (2021)  Decision making in the end-of-life care of patients who are terminally ill with cancer – a qualitative descriptive study with a phenomenological approach from the experience of healthcare workers  Colombia | Share decision making  To explore what aspects determine decision-making at the End of Life to identify difficulties and promote strategies to facilitate conversations, advance care planning and decision-making in Colombia. | Qualitative phenomenology study | Semi-structured interviews with a range of health care professionals (N=28) all involved in EoL care of cancer patients in three hospitals in Colombia. | Phenomenological approach and inductive coding | Three main conditions influence the SDM process: patients clinical and socio-cultural conditions, physician’s relationship with patients and caregivers and palliative care education, and health system possibilities and limitations. | **Applying a holistic approach**   - Having Empathy: the ability to recognize and understand patients emotions - Time investment: leads to greater knowledge of the patient’s life, builds trust, opens a comfortable space to express patients own ideas regarding management options and initiate Eol discussions - Adapt to the culture of a person à requires special skills in management of any diversity   **Communication and active listening**   - Having Empathy: the ability to recognize and understand patients emotions   **Managing time effectively**   - Time investment: leads to greater knowledge of the patient’s life, builds trust, opens a comfortable space to express patients own ideas regarding management options and initiate Eol discussions |
| Oerlemans et al. (2021)  Learning shared decision-making in clinical practice  Netherlands | To explore how shared decision-making (SDM) is learned in clinical practice according to professionals and patients. | Qualitative | Focus group and individual interviews with interns (n = 9), residents (n = 12), senior physicians (n = 13), and (former) patients and relatives (n = 13) in fertility care and intensive care. | Thematic content analysis | Learning SDM is a complicated task for both students and professionals in healthcare. Relevant factors are the involvement of patients, the role of informal learning processes and role models, and the importance of reflective practice. | **Applying a holistic approach**   - Attune to the level of patients and relatives   **Communication and active listening**   - Strategically employ silence - “not beat around the bush” - Show empathy - “Attentive to both patients and relatives’   **Reflecting and self-reflection**   - “Checks whether patient and relative have understood what was explained” - Self-reflection/transparent: “do they deliver on their promises, are they easily accessible, aware of their limits (and honest about them), consult other professionals when necessary, and how rigidly the adhere to guidelines” |
| Yu et al. (2022)  Determining the Development Strategy and Suited Adoption Paths for the Core Competence of Shared Decision-Making Tasks through the SAA-NRM Approach.  Taiwan | To explore the critical factors of SDM  competence and the interaction relation between aspects and further design appropriate  development strategies. | Mixed-methods | Questionaries with physicians (N=139), expert interviews (n=4), literature search | SAA (satisfaction-attention analysis) | Precise quantiﬁcation and network architecture will be helpful in increasing the consensus between the key elements in SDM competency development from physicians’ perspectives. Furthermore, physicians’ professionalism in performing SDM tasks may reduce patients’ uncertainty and anxiety, strengthen self-efﬁciency and trust, and  diminish the barriers to SDM | **Applying a holistic approach**   - Adapt to socioeconomic cultures, backgrounds and personality traits and their families and give empathic responses   **Communicating and active listening**   - Have familiarity with reviewing evidence from literature and applying them appropriately to support SDM - Avoid specialized terms - Use simple and familiar words - Provide an appropriate amount of information - Clarify myths - Emphasize a crucial information - Communicate verbal and non-verbal - Develop multiple-choice options - Provide all the necessary information: advantages, disadvantages, analysing risks - Give enough time for the patient to think - EBM framework: ask, acquire, appraise, apply and assess   **Managing time effectively**   - Using a decision aid - Teaming with multi-disciplinary members   **Encouraging patient participation**   - Clarify the patient’s fundamental role in SDM - Notify that a decision is to be made - “Encourage patient participations, guide patients to express personal opinions, improve patient self-efficacy, and respect for autonomy”   **Reflecting and self-reflection**   - Guide the patient to reply information to check their understanding - Arrange follow-up |
| Hayes et al. 2018  Barriers and facilitators to shared decision making in child and youth  mental health: clinician perspectives using the Theoretical Domains  Framework  United Kingdom | To explore clinicians’  views of the barriers and facilitators around SDM with  young people who have internalising disorders along with  their parents, within two services in England. | Qualitative | Face to face interviews (n=5), telephone interviews (n=5) and focus groups (n=5 site B).  Two site’s. One NHS Trust (Site A) consisted  of a single child and adolescent mental health service  (CAMHS) based in the east of England. The second NHS  Trust consisted of four CAMHS clinics based in the Midlands. | Thematic analysis using braun and clark. Kappa was used to determine a good level of agreement | Barriers and skills have been found affecting SDM, both with young people who have internalising  difficulties and their parents. These  included skills such as listening, the young person having  limited capacity due to mental illness, not knowing what  options are available, and finite resources. Novel findings included: containment as a skill, clinician uncertainty over  the term SDM, the use of team members to help offer suggestions  when the clinician was stuck, and for one clinician  a concern that SDM could make existing difficulties worse. | **Applying a holistic approach**   - Being open, honest, transparent and listening to young people and parents - Take into account their preferences and cultural values   **Communication and active listening**   - Basics is listening to be more involved and connect with people   **Managing time effectively**   - Review sometimes not completed on time only on ad hoc basis - Lack of appropriate room, results in lack of space, having limited time for SDM - Assessment process takes time, less time for SDM - Busy to try to keep to time, leads to directive decision making   **Encouraging patient participation**   - Responsible for outlining the different options - Only suggest options that are available (🡪 hereby not interrupting built relationship or make therapy more challenging) - SDM gives empowerment, a sense of autonomy and control - Be informed in order to feel more comfortable during SDM - Negotiations and containment, two steps forward and four steps back   **Reflecting and self-reflection**   - Reviewing treatment progress and goals to understand how the person is progressing |
| Beek-Peters et al. (2021)  Professionals’ views on shared decision-making  In severe aortic stenosis  Netherlands | To provide insight into  professionals’ perceptions of and experiences with  SDM in the treatment of symptomatic patients with  severe AS. | Qualitative | Semi structured interviews to explore  in depth perceptions and experiences  21 interviews  eight cardiothoracic surgeons, seven interventional  cardiologists, five nurse practitioners and one physician assistant  The 21 professionals represented 9 out of 16 heart  centres in the Netherlands.  9 face-to  face and 12 online interviews | Thematic analysis | Not all professionals  consider patient participation as an element of SDM:  some valued and even expected patient participation in deciding  on the treatment, while others did not attribute a very active role  to the patient. Generally, professionals considered patient knowledge  and their opinion of treatment to be essential for SDM | **Applying a holistic approach**   - Knowing patient characteristics for SDM, such as low level of educating, difficulties in communication   **Communication and active listening**   - Written and digital information regarding options increases patients knowledge - Use verbal strategies during consultation to improve knowledge, clarifying aim and risks, ask for patients knowledge and opinion about treatment - Building trust by communication techniques such as showing empathy and directing conversation and moderating   **Managing time effectively**   - Implementing SDM is too time consuming and time for consultations is limited   **Encouraging patient participation**   - SDM gives patient support for the decision - Some emphasised that patients have to make the final decision, while others added that professionals can direct the decision during consultations - “you have to try to explain why you make that choice for a patient” - SDM guided patients towards empowerment of their own health situation - Decision aids were not familiar to improve patients’ knowledge |
| Zeuner et al. (2014)  Physicians’ perceptions of shared decision-making  behaviours: a qualitative study demonstrating the  continued chasm between aspirations and clinical  practice  America | To explore clinicians’ attitudes, beliefs  and perceived social norms about SDM  communication behaviours | Qualitative | Semistructured,  qualitative interviews with physicians  . With physicians (n=20) in five practice areas: obstetrics and  gynaecology, internal medicine, emergency  medicine, medical oncology and surgery | Thematic analysis | Our research suggests that physicians perceive  a number of individual, cultural and systemlevel  obstacles to engaging in SDM communication  behaviours. | **Applying a holistic approach**   - “communicating in a language they will understand and going into as much depth as they’re willing. . .involving them in their care helps them to make a decision they feel is most appropriate for them and their lifestyle”   **Communication and active listening**   - approve of acknowledging decisions to patients, that is discussing multiple options with the patient and facilitating the decision-making process though effective communication   **Managing time effectively**   - Time and the specific clinical setting were identified as barriers to engaging in SDM. Even when participants supported the idea of engaging patients in SDM, many noted that lack of time can make these discussions more difficult to have - I think a lot of times when you are strapped for time. . .you don’t have time to discuss what the risks and benefits of the option are - Increasing patients’ access to effective decision support interventions that can be used outside of the consultation could prepare patients for SDM discussions without adding time to an already time-pressured visit   **Encouraging patient participation**   - “there has been a shift in the culture of medicine I believe to engage the patient more in decision making” - concerned about explaining the concept of equipoise to patients, fearing that patients would interpret this as a sign of physicians’ incompetence and not as indicative of the state of the scientific evidence - they felt that patients with less education might feel overwhelmed by engaging in SDM. - it seems like they have had a pretty good level of understanding, they read up or they seem educated on the situation, it’s easier to discuss options with them. - explore patients’ decision role preferences, describing clinical equipoise and providing an overview of why patients are invited to participate in decision making. Next, they could explicitly ask patients about their desired role in decision making |
| Kannan, et al. (2020)  Surgeons’ Views on Shared Decision-Making  America | Explored surgeons’ attitudes and beliefs about SDM t | Qualitative | Semi-structured interviews  Both academic and non-academic surgeons (n=18) were recruited from various surgical specialties in Baltimore. | Conventional thematic content analysis | We found that surgeons were broadly supportive of patient engagement through SDM and believed that SDM improved patient education and empowerment. Nevertheless, surgeons believed that SDM was only appropriate for a subset of patients and treatment decisions, based on intuitive assessments | **Applying a holistic approach**   - “I think all patients are different, and I think some of them come to a surgeon wanting them to tell them what to do, so I am not sure [SDM] is the right thing for all patients”   **Communication and active listening**   - I’ve been around long enough to realize how much in-depth I can get with someone and if somebody looks particularly anxious….. …..so in that case I may try to keep it simple*.”*   **Managing time effectively**   - education resource with their patients, including websites (both academic and nonacademic), pamphlets, books, diagrams, and others - Only a small subset of surgeons used formal decision aids with their patients during decision-making   **Encouraging patient participation**   - “they have the best handle on discerning their values so they can make the decision. … And it’s good to empower patients” - surgeons highlighted patient involvement as enabling patients to be more informed about their care - downside to involving patients in decision-making was the potential for overwhelming or confusing patients. - Some surgeons with clear preferences also sought to persuade patients with conflicting preferences to change their minds - In other cases, surgeons only disclosed their preferences when prompted by patients - Often, patient desire to participate in decision-making is not fixed but rather evolves through the building of trust and partnership through a strong patient-physician relationship. |
