# Supplementary material for: Skills to act from a Positive Health approach: in comparison with shared decision-making: a scoping review
Source: Front Public Health. 2025 May 2;13:1530427. doi: 10.3389/fpubh.2025.1530427 (PMC12081412; doi:10.3389/fpubh.2025.1530427)
Supplement: Supplementary file 3 [file Table_3.docx]

| Qualitative PH Studies |  | |  | | Textual evidence:  Narrative | | |
| --- | --- | --- | --- | --- | --- | --- | --- |
| Quality Criteria | [38] | [37] | [11] | Quality Criteria | | [39] | |
| Philosophical perspective and research methodology | U | U | Yes | Generator credible or appropriate source | | | Yes |
| Research methodology and research objectives | Yes | Yes | Yes | Relationship between text and context | | | No |
| Research methodology and methods for data collection | Yes | Yes | Yes | Logical sequence used | | | Yes |
| Research methodology and methods for data analysis | Yes | Yes | Yes | Similar conclusion drawn as the narrator | | | Yes |
| Research methodology and results interpretation | Yes | Yes | Yes | Conclusions flow from narrative account | | | Yes |
| Locating the researcher culturally or theoretically | U | No | Yes | Is this account a narrative | | | Yes |
| Influence of the researcher | Yes | No | Yes |  | | |  |
| Participants adequately represented | U | Yes | Yes |  | | |  |
| Ethical approval | No | Yes | Yes |  | | |  |
| Conclusions flowing from data | Yes | Yes | Yes |  | | |  |

*Table 1: critical appraisal of included PH studies. Yes = information available, No = information not available, U = unclear. This table is based on the critical appraisal tool for qualitative studies and narratives from Joanne Brigss institute[15]*

| Qualitative Studies |  | Studies | | | | | | | | Randomized controlled trial | Study | Cross sectional studies | | Study |
| --- | --- | --- | --- | --- | --- | --- | --- | --- | --- | --- | --- | --- | --- | --- |
| Quality Criteria | [26] | [35] | [27] | [29] | [28] | [34] | [31] | [30] | [32] | Quality criteria | [33] | Quality criteria | [36] | |
| Philosophical perspective and research methodology | U | U | Yes | U | U | U | U | U | U | True randomization used | Yes | Criteria for inclusion defined | Yes | |
| Research methodology and research objectives | Yes | Yes | Yes | Yes | Yes | Yes | Yes | Yes | Yes | Allocation groups concealed | Yes | Study subjects and setting described in detail | U | |
| Research methodology and methods for data collection | No | Yes | Yes | Yes | U | Yes | Yes | Yes | Yes | Baseline groups similar | Yes | Exposure measured valid and reliable | Yes | |
| Research methodology and methods for data analysis | No | Yes | Yes | Yes | No | Yes | Yes | Yes | Yes | Participants blind to assignment | Yes | Objective standard criteria used for measurement | Yes | |
| Research methodology and results interpretation | Yes | Yes | Yes | Yes | Yes | Yes | Yes | Yes | Yes | Those delivering treatment blind to assignment | Yes | Confounding factors identified | Yes | |
| Locating the researcher culturally or theoretically | No | Yes | Yes | U | No | No | U | No | No | Outcomes assessors blind to assignment | No | Strategies for confounding | No | |
| Influence of the researcher | No | U | Yes | U | No | U | U | U | No | Groups treated identically | Yes | Outcomes valid and reliable | Yes | |
| Participants adequately represented | No | Yes | Yes | Yes | No | Yes | Yes | Yes | Yes | Follow up complete | No | Appropriate statical analysis | Yes | |
| Ethical approval | Yes | U | Yes | U | Yes | U | Yes | U | U | Participants analysed in their randomized group | U |  |  | |
| Conclusions flowing from data | Yes | Yes | Yes | Yes | Yes | Yes | Yes | Yes | Yes | Outcomes measured the same for treatment groups | Yes |  |  | |
|  |  |  |  |  |  |  |  |  |  | Outcomes measured reliable | Yes |  |  | |
|  |  |  |  |  |  |  |  |  |  | Appropriate statical analysis | Yes |  |  | |
| *Table 2: critical appraisal of included SDM studies. Yes = information available, No = information not available, U = unclear. This table is based on the critical appraisal tool for qualitative studies, randomized controlled trials and cross-sectional studies from Joanne Brigss institute[15]* |  |  |  |  |  |  |  |  |  | Trail design appropriate | Yes |  |  | |
